# Glucose metabolism-related gene polymorphisms as the risk predictors of type 2 diabetes

**DOI:** 10.1186/s13098-020-00604-5

**Published:** 2020-11-04

**Authors:** Cuilin Li, Yuping Yang, Xin Liu, Zhongyu Li, Hong Liu, Qiuhong Tan

**Affiliations:** 1 Department of Pharmacy, The Affiliated Zhuzhou Hospital Xiangya Medical College CSU, Zhuzhou, 412007 Hunan China; 2 Laboratory Medical Center, The Affiliated Zhuzhou Hospital Xiangya Medical College CSU, Zhuzhou, 412007 China; 3 Department of Metabolism and Endocrinology, The Affiliated Zhuzhou Hospital Xiangya Medical College CSU, Zhuzhou, 412007 China

**Keywords:** Type 2 diabetes mellitus, Genetic polymorphism, G6PC2, GCK, GCKR, OCT3

## Abstract

Type 2 diabetes mellitus (T2DM) is a complex polygenic metabolic disease characterized by elevated blood glucose. Multiple environmental and genetic factors can increase the risk of T2DM and its complications, and genetic polymorphisms are no exception. This review is mainly focused on the related genes involved in glucose metabolic, including *G6PC2*, *GCK*, *GCKR* and OCT3. In this review, we have summarized the results reported globally and found that the genetic variants of *GCK* and OCT3 genes is a risk factor for T2DM while *G6PC2* and *GCKR* genes are controversial in different ethnic groups. Hopefully, this summary could possibly help researchers and physicians understand the mechanism of T2DM so as to diagnose and even prevent T2DM at early time.

## Background

Diabetes is one of the major chronic disease threatening human health. There were 451 million people with diabetes worldwide up to 2017. And it was estimated that in 2045 the number of diabetes patients will increase to 693 million [1]. In China, the estimated numbers of Type 2 Diabetes Mellitus (T2DM) was 113.9 million, representing 11.6% of Chinese population [2]. T2DM is a lifelong disease characterized by hyperglycemia, showing with drinking more, eating more, peeing more and losing weight. If the glucose is not efficiently controlled, the patients will have more chance to develop complications, such as nephropathy, peripheral neuropathy, diabetic retinopathy, amputation, vascular disease, heart disease and stroke. These complications ultimately decrease the quality of life, increase the economic burden of patients [3]. T2DM can be caused by various factors, including obesity, physical inactivity, family history, hypertension and age [4]. Apart from these, genetic factors are considerable since many genes and their interactions play important roles in the development of T2DM [5], such as *PRKAA2* [6], *ABCA1* [7], *FTO* [8], *FADS* [9] and *TCF7L2* [10]. Therefore, finding and summarizing the gene variants among different ethnic groups will be helpful to understand the treatment, prevention and complications of T2DM.

## Candidate genes for T2DM

**Fig. 1:**
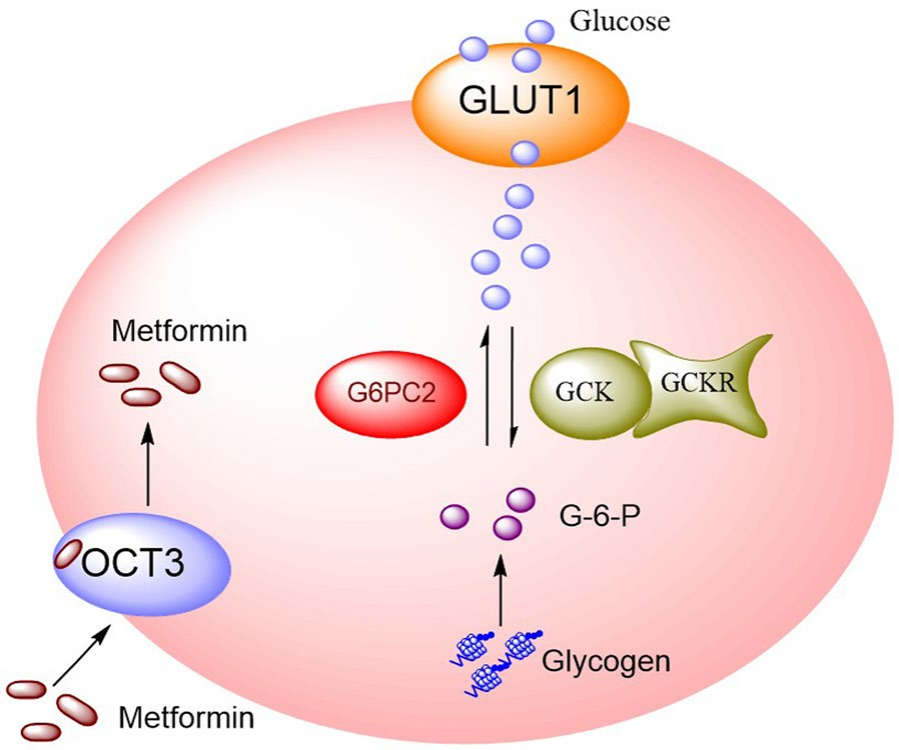
Glucose metabolism-related gene involving in glucose regulation. Glucose is taken up into the hepatic cell via glucose transporters 1 (GLUT1). In the process of glycogenolysis, liver glycogen is hydrolyzed and isomerized to glucose-6-phosphatase (G-6-P). Then glucose-6-phospatase catalytic subunit 2 (G6PC2) catalyzes the production of free glucose to maintain blood glucose balance, and this process is also the key step of gluconeogenesis. In the process of glucose activation, glucokinase (GCK) and glucokinase regulator (GCKR) is the key enzyme to regulate glucose phosphorylation, which followed by glycolysis and aerobic oxidation. At the same time, organic cation transporter 3 (OCT3) is a transporter of metformin, which involved in the regulation of HbA1c levels and glucose

People with family history of diabetes will have 2–4 times higher risk to develop T2DM than the unrelated individuals [11]. With the development of pharmacogenomics, more and more genetic variants were reported to associated with the susceptibility and treatment of T2DM. As we all know, single nucleotide polymorphism (SNP) is one of the main forms of genetic variation, which can affect the expression of glucose metabolism-related gene. Glucose metabolism-related gene is involved in glucose regulation (Fig. 1), thus affecting the susceptibility of T2DM. In this review, we will focus on *G6PC2*, *GCK*, *GCKR*, and *OCT3* genes and their association with the susceptibility of T2DM as shown in Table 1. The products of these genes are related to the biochemical pathway leading to T2DM or higher blood glucose. The enzyme encoded by *G6PC2* belong to the glucose-6-phosphatase catalytic subunit family, which is involved in the terminal step in gluconeogenic and glycogenolytic pathways, allowing the release of glucose into the bloodstream. The product of *GCK* is responsible for regulating glucose and the secretion of insulin. Products of *GCKR* inhibits glucokinase in liver and pancreatic islet cells by binding non-covalently to form an inactive complex with the enzyme. *OCT3*, also named *SLC22A3*, is critical for elimination and transportation of many endogenous small organic cations as well as a wide array of drugs and environmental toxins.

**Table 1:** Summarize of *G6PC2*, *GCK*, *GCKR*, and *OCT3* gene polymorphism with T2DM in various ethnic groups

Gene	Polymorphism	Significance	OR	Case/Control	Ethnicity	References
G6PC2	rs492594 G > C	Y	0.57	538/538	Han Chinese	[12]
	rs492594 G > C	Y	1.14	1876/1800	Eastern Han Chinese	[13]
	rs492594 G > C	Y	1.70	185/377	RIYADH COHORT	[14]
	rs13387347 T > C	Y	1.17	1876/1800	Eastern Han Chinese	[13]
	rs13387347 T > C	N		538/538	Han Chinese	[12]
	rs16856187 A > C	Y	1.19	1876/1800	Eastern Han Chinese	[13]
	rs16856187 A > C	N		538/538	Han Chinese	[12]
	rs2232316 G > A	N		1876/1800	Eastern Han Chinese	[13]
	rs2232328 C > G	Y	1.64	185/377	RIYADH COHORT	[14]
GCK	rs4607517 T > C	Y	1.20	853/3210	American Indians	[15]
	rs1476891 A > G	Y	1.26	1658/1946	Pima Indians	[15]
	rs55714218 G>-	Y	0.84	853/3210	American Indians	[15]
	rs1799884 G > A	Y	1.12	2628/2041	Netherlands	[16]
	rs1799884 G > A	Y	1.23	1244/3189	French	[17]
	rs1799884 G > A	Y	1.24	1193/1055	Moroccans	[18]
GCKR	rs780094 G > A	Y	1.78	538/538	Han Chinese	[12]
	rs780094 G > A	Y	0.67	424/1884	Han Chinese	[19]
	rs780094 G > A	N		736/768	Northern Han Chinese	[20]
	rs780094 G > A	Y	0.71	488/398	Japanese	[21]
	rs780094 G > A	Y	1.22	1118/1161	Han Chinese	[22]
	rs1260326 C > T	Y	0.74	424/1884	Han Chinese	[19]
	rs1260326 C > T	N		538/538	Han Chinese	[12]
	rs2293572 C > G	N		538/538	Han Chinese	[12]
	rs3817588 A > G	Y	1.24	1118/1161	Han Chinese	[22]
OCT3	rs3088442 G > A	Y	0.02	150/152	Iran	[23]
	rs2292334 G > A	Y	2.76	150/152	Iran	[23]

## Glucose-6-phosphatase catalytic subunit 2 (*G6PC2*) gene

*G6PC2* is part of the glucose-6-phosphatase catalytic subunit family, which can catalyze the hydrolysis of glucose-6-phosphate, allowing the release of glucose into the bloodstream. *G6PC2* is located at 2q31.1 on human chromosome with 4 exons. This gene encodes a 355 amino acid protein which is a negative regulator of basal glucose-stimulated insulin secretion. The deletion of G6PC2 in pancreatic islet beta cell was reported to reduce fasting blood glucose [24]. GWAS and mouse studies suggested that single nucleotide polymorphisms in *G6PC2* gene were associated with variations in fasting blood glucose (FBG) but not fasting plasma insulin [25]. A research in Science reported that SNP rs560887 in *G6PC2* gene was associated with FBG and pancreatic beta cell function but not associated with T2DM risk in three populations [26]. A study on Europeans shown that *G6PC2* rs560887, rs2232316 and rs13431652 were potentially causative SNPs of elevated FBG level [27]. A low-frequency and rare exome chip found that G6PC2 rs138726309 (H177Y), rs2232323 (Y207S), rs146779637 (R283X) and rs2232326 (S324P) were associated with FG [28]. Further, various studies suggested that the polymorphisms and haplotypes in *G6PC2* gene were associated with susceptibility of T2DM [29]. In Chinese population, Li et al. verified that the C allele of rs780094 and the GC genotype of rs492594 were significantly associated with the increased risk of T2DM. Also, this research found *G6PC2* and *GCKR* haplotypes were associated with the susceptibility of T2DM [12]. Apart from this, another research also demonstrated the relationship between rs492594 and T2DM risk. *G6PC2* rs16856187 was shown as the strongest evidence for the association with T2DM [13]. However, the results seem controversial in Chinese population. While another study found no significant association between rs16856187 and T2DM risk [12]. Maybe this discrepancy is the results of the interaction of gene-region or gene-environments. After all, China is a vast country with huge variation on geography. Rs2232328 and rs492594 were also reported to influence the susceptibility of T2DM in Arabian [14].

## Glucokinase (*GCK*) gene

Glucokinase (*GCK*) can catalyze the phosphorylation of glucose to glucose-6-phosphate. In pancreatic β-cell, it plays a significant role in regulating glucose metabolism and insulin secretion [30]. Therefore, it is understandable that the mutation or polymorphism of *GCK* gene can cause pathoglycemia and diabetes mellitus. It is reported that mutation of *GCK* is associated with Chinese MODY (maturity onset diabetes of young type) [31]. The meta-analysis shown that the polymorphisms of GCK rs1799831 was associated with gestational diabetes mellitus (GDM) in Indian population [32]. Genetic polymorphism in *GCK* gene has been shown to be associated with the susceptibility of T2DM. 3’UTR SNP, chr7:44,184,184-G/A in GCK was reported to influences the rate of oxidation of carbohydrate, 24 h energy expenditure and diabetes risk in Pima Indians. Compared with individuals with A allele, individuals with G allele had lower rate of oxidation of lipid and higher 24 h energy expenditure (by 520 kJ/day) [15]. SNP rs1276891 and chr7:44,184,184 3′UTR in *GCK* were associated with T2DM in American Indian [15]. A case-control study in Netherlands revealed weak evidence for an association between rs1799884 and T2DM [16]. In French, *GCK* rs1799884 was found to increase risk of T2DM [17]. A meta-analysis involved of 24 studies also reported that rs1799884 was associated with the susceptibility of T2DM and the regulation of impaired glucose. Further, this meta-analysis found significant increase of fasting plasma glucose level in rs1799884 A allele compared with G allele [33]. Another study in Moroccans shown significant association of *GCK* rs1799884 polymorphism with T2DM [18]. In Japanese, *GCK* rs4607517 was deemed to be associated with HbA1c level, but not associated with the susceptibility of T2DM [34].

## Glucokinase regulator (*GCKR*) gene

*GCKR*, also known as *GKRP*, encodes a protein belonging to the GCKR subfamily of the Sugar Isomerase family of proteins. The *GCKR* is mainly expressed in liver. is a regulatory protein that inhibits glycolysis, glycogen deposition, and de novo lipogenesis by binding to the glucokinase and impairing it [35]. The mutation or gene variants of *GCKR* was reported to be associated with several clinical manifestation, such as T2DM [36], nonalcoholic fatty liver disease (NAFLD) [37], familial combined hyperlipidemia (FCHL) [38], coronary artery disease, ischemic stroke [39], gout [40] and chronic kidney disease [41]. A number of studies have shown the significant association of GCKR polymorphism with T2DM in different ethnic groups. In Han Chinese population, *GCKR* rs780094 A allele was reported to be associated with decrease risk of T2DM and obesity. Gene-gene interaction was found to influence fasting glucose between *GCKR* rs780094 and *GCK* rs1799884. The influence of *GCKR* rs1260326 polymorphism on T2DM was also verified in this study [19]. The same results that rs780094 associated with T2DM in Chinese population was also reported by Li et al. [12]and Gao et al. [22]. In Japanese, the results of multiple regression analysis shown that rs780094 was a marker of T2DM susceptibility [21]. In contrast, some studies have also shown no association between *GCKR* rs780094 and T2DM [20]. Apart from T2DM, polymorphisms of *GCKR* was also a susceptibility gene of gestational diabetes. Some studies reported that GCKR rs780094 was associated with the susceptibility of gestational diabetes in Malaysian population [42] and Brazilian [43].

## Organic cation transporter 3 (OCT3) gene

*OCT3*, also known as *SLC22A3*, located at 6q25.3 on human chromosome with 15 exons. *OCT3* is a polyspecific organic cation transporter mainly expressed in the liver, kidney and intestine. *OCT3* is contributed to transfer many endogenous small molecules, drugs and environmental toxins [44]. Therefore, mutations and variants in *OCT3* will influence the development of various disease and the efficacy of multiple drugs. SNPs in *OCT3* have been shown to be related to diverse conditions, including lipoprotein(a) concentration, cardiovascular disease [44], colorectal cancer [45], metformin pharmacokinetics, esophageal cancer [46], pancreatic cancer [47], and T2DM [23]. In Iranian, *OCT3* rs3088442 G > A was reported to be a protective factor of T2DM, while rs2292334 to be a risk factor of T2DM [23]. As a drug transporter, there were more studies reported the relationship between polymorphisms of *OCT3* and metformin. Wang et al. hold the view that the absolute bioavailability of metformin in oct3+/+ mice was significantly increased compared with the oct3-/- mice [48]. Another study reported that *OCT3* played an important role in the absorption and elimination of metformin in mice [49]. In Korea, *OCT3* haplotype was reported to influence the pharmacokinetics of metformin [50]. And *OCT3* was responsible for metformin accumulation and secretion in salivary glands [51]. In T2DM patients, the mean reduction in HbA1c levels was higher in patients with OCT3 rs2292334 A allele than in those with the homozygous G allele [52]. In Pakistani population, the A allele of *OCT3* rs3088442 was a protective factor and associated with clinical efficacy of metformin [53].

## Conclusions

With the development of living standard, the incidence of diabetes is increasing rapidly around the world. This urges us to identify the high-risk individuals at an early stage so as to prevent or put off the development of diabetes. As we all known, diabetes is a disease resulted from many factors and their interactions, including environment, eating habits, lifestyle, ethnicity, and family history. Apart from these, genetic factors also play an important role in the occurrence of diabetes. And genetic factors also interact with environment to induce the individuals to diabetes. This is the reason why a susceptibility gene might show different phenotype in different populations or regions. Various studies have reported the association between genetic variants and the susceptibility of T2DM. In this manuscript, we summarized the results on the association of *G6PC2*, *GCK*, *GCKR* and *OCT3* genes with T2DM from various global studies. And hope this review could possibly give a better perspective to help researchers understanding the pathogenesis of T2DM. Results verify the polymorphisms of *GCK* and *OCT3* genes have potential effect on T2DM whereas the relationship between *G6PC2*, *GCKR* and T2DM susceptibility is controversial in different studies. The difference in ethnicity and environment may account for this discrepancy. Nevertheless, further research that investigate their role in T2DM is required in more and larger groups globally. Only in this way we can understand the biological and molecular mechanism of T2DM thoroughly, and we can find out more precise biomarker to identify at-risk patients in early stage.

## Data Availability

Not applicable.
